# Acute-on-chronic liver failure due to hepatitis B

**DOI:** 10.3389/fgstr.2023.1016915

**Published:** 2023-03-09

**Authors:** Pallavi Garg, Kaushal Madan

**Affiliations:** Centre of Gastroenterology, Hepatology and Endoscopy, Max Super Specialty Hospital, Saket, New Delhi, India

**Keywords:** HBV, ACLF (acute on chronic liver failure), plasma exchange, Nuc, HBV reactivation

## Abstract

Acute-on-chronic liver failure (ACLF) is a complex health problem with a high short-term mortality. It is a form of end-stage liver disease (ESLD) characterized by acute hepatic insult on the background of an underlying chronic liver disease leading to other extrahepatic organ failures. Due to its rapid rate of progression, it is a challenge for both hepatologists and intensivists to treat. Many variations exist regarding its definition, leading to descriptions of various clinical phenotypes. Patients who have chronic hepatitis B (CHB) or Hepatitis B virus (HBV)-related cirrhosis are also prone to develop hepatic or extrahepatic failures when they develop a superadded insult. Different severity criteria and prognostic scores have been proposed and validated among various populations and various etiologies including HBV. The management mainly focusses on support of various organ failures while waiting for the liver to regenerate, for liver transplantation, or, in the case of HBV-related ACLF (HBV-ACLF), for the anti-virals to take effect. Liver transplantation still remains the definitive treatment for HACLV in general and even for HBV- ACLF. Medical therapies, such as nucleos(t)ide analogue (NUCs)and artificial liver support, may improve survival in a subset of patients with HBV-related ACLF. This review updates the understanding of HBV-ACLF from epidemiological and clinical studies and provides new insights into the definition, diagnostic criteria, epidemiology, pathogenesis, treatment, and prognostication of HBV-ACLF.

## Introduction

Around 350 million people globally are chronically infected with hepatitis B virus; 75% of these cases are in the Asia-Pacific region alone, where CHB may be the leading cause of liver disease-related mortality (1).The disease spectrum of HBV varies from inactive carrier (now also called e- minus chronic HBV infection) to CHB, cirrhosis, hepatocellular carcinoma, and liver failure. Chronic infection with hepatitis B can be divided in to four phases based on the interaction of host immune response with the virus and virally infected hepatocytes. These are: e-positive chronic HBV infection, e-positive chronic hepatitis B, e-negative chronic HBV infection, and e negative chronic hepatitis B.

Studies suggest that 15-37% of patients with HBV infection have spontaneous acute exacerbations within 4 years (2). Some of these patients develop liver failure during such exacerbations and are labelled as acute-on-chronic liver failure; the mortality rate for these patients is reported to be as high as 30%-70% (3, 4). In the Asia-Pacific region, Hepatitis B appears to be the most common cause of ACLF. In China, 80% of ACLF is due to hepatitis B (5) and reactivation alone as the acute hepatic insult is found to be the cause in 59% of cases.

## Definition of ACLF

There is no universal consensus on the definition of ACLF and different groups have come up with different concepts. The Asian Pacific Association for the study of the Liver (APASL) provided the first definition in 2009. It defined ACLF as “an acute hepatic insult manifesting as jaundice and coagulopathy, complicated within 4 weeks by ascites and/or encephalopathy in a patient with previously diagnosed or undiagnosed chronic liver disease or cirrhosis” (3). In 2014, it was modified to include high 28 day mortality (6).

In 2011, at the EASL-AASLD single topic symposium, ACLF was defined as: “acute deterioration of pre-existing liver disease following a precipitating event and an association with increased short-term mortality because of multisystem organ failure” (7).

Later in 2013, the EASL chronic liver failure (CLIF) consortium group released the first result of the ‘EASL-CLIF Acute-on-Chronic Liver Failure in Cirrhosis (CANONIC) study’, which proposed the first evidence-based ACLF definition (4). In the CANONIC study, ACLF was identified (among cirrhotic patients with acute decompensation) as multi-organ failure(s) (defined by the CLIF-OF score) and a predefined mortality of 15%. The CANONIC criteria also includes patients with underlying decompensated cirrhosis. The following events were considered as acute decompensating events: ascites, hepatic encephalopathy (HE), gastrointestinal bleeding, and/or bacterial infections.

The World Gastroenterology Organization (WGO) tried to combine the EASL and APASL criteria to reach a working definition: “ ACLF is a syndrome in patients with chronic liver disease with or without previously diagnosed cirrhosis characterized by acute hepatic decompensation resulting in liver failure (jaundice and prolonged international normalized ratio(INR)) and one or more extra hepatic organ failure associated with increased mortality within a period of 28 days to 3 months from the onset” (8).

NACSELD (North American Consortium for the Study of End-Stage Liver Disease) defined ACLF as ≥ 2 organ failures in hospitalized patients with cirrhosis (9). A recent guideline by ACG defines ACLF as “a potentially reversible condition in patients with chronic liver disease with or without cirrhosis that is associated with the potential for multiple organ failure and mortality within 3 months in the absence of treatment of the underlying liver disease, liver support, or liver transplantation.” (10).

### HBV-ACLF definition

HBV-related ACLF has earlier been defined as per the previously mentioned definitions for ALCF in general. For this reason, it is difficult to ascertain if these definitions work well for pure HBV-ACLF or not. HBV-ALCF, as is evident from the descriptions of researchers who have included only HBV-infected patients with ACLF, appears to be different from the ACLF in general, in terms of the precipitants, underlying disease, and natural course; it therefore merits a separate discussion, if not a separate identity.

The Chinese Group on the Study of Severe Hepatitis B-ACLF (COSSH-ACLF) defined HBV-ACLF as a complicated syndrome with a high short-term mortality that develops in patients with HBV-related chronic liver disease regardless of the presence of cirrhosis, is characterized by acute deterioration of liver function and hepatic and/or extrahepatic organ failure (11). The COSSH- ACLF group suggests that patients of chronic hepatitis B with or without liver cirrhosis with total bilirubin ≥ 12mg/dl and INR ≥ 1.5 should be diagnosed as ACLF. This could diagnose 20% more patients, thus increasing their opportunity to receive timely appropriate therapy. They found these criteria to be significantly more sensitive than the EASL-ACLF criteria for diagnosing patients with HBV ACLF. (**Table 1**)

**Table 1:** Criteria for the diagnosis of ACLF.

	European Association for the Study of the Liver - Chronic Liver Failure (EASL-CLIF) Consortium	North American Consortium for the Study of End-stage Liver Disease (NACSELD)	Chinese Group on the Study of Severe Hepatitis B (COSSH)	Asian-Pacific Association for the Study of the Liver (APASL) ACLF Research Consortium (AARC)
Type of study	Original article of CANONIC study, prospective, observational study in patients with cirrhosis in Europe	Original article patients in USA and Canada	Original article COSSH study, prospective, observational in patients with cirrhosis or severe liver injury due to chronic hepatitis B in China	Consensus document involving international experts from the APASL, published in 2009 and updated in 2014 and 2019
Patients considered in the definition	Compensated and decompensated cirrhosis	Patients with acutely decompensated cirrhosis, with or without prior episode(s) of decompensation	Patients with acute decompensation of HBV-related chronic liver disease, with or without cirrhosis	Compensated cirrhosis. Non-cirrhotic CLD
Precipitating disorders	Intrahepatic (alcoholic hepatitis), extrahepatic (infection, gastrointestinal hemorrhage), or both	Extrahepatic (infection)	Intrahepatic (HBV reactivation), extrahepatic (bacterial infection), or both	Intrahepatic
Major organ systems considered for the definition	6: liver, kidney, brain, coagulation, circulation, and respiration	4: kidney, brain, circulation, and respiration. Liver and coagulation are not considered	6: Liver, kidney, brain, coagulation, circulation, and respiration	Liver dysfunction is central to the definition; hepatic encephalopathy may be present as a consequence
Definition and stratification of ACLF	ACLF is divided into three grades of increasing severity. ACLF grade 1 includes three subgroups: − patients with single kidney failure – patients with single liver, coagulation, circulatory, or lung failure that is associated with creatinine levels ranging from 1.5 mg/dl to 1.9 mg/dl or hepatic encephalopathy grade 1 or grade 2, or both − patients with single brain failure with creatinine levels ranging from 1.5 mg/dl to 1.9 mg/dl. ACLF grade 2 includes patients with two organ failures. ACLF grade 3 includes patients with three organ failures or more	Patients are stratified according to the number of organ failures: 2, 3, or all 4 organ failures, respectively	Three grades of increasing severity. ACLF grade 1 includes four subgroups: − patients with single kidney failure − patients with single liver failure and either an INR of 1.5 or more, creatinine levels ranging from 1.5 mg/dl to 1.9 mg/dl, hepatic encephalopathy grade I or II, or any combination of these alterations − patients with a single type of organ failure of the coagulation, circulatory, or respiratory systems and either creatinine levels ranging from 1.5 mg/dl to 1.9 mg/dl, hepatic encephalopathy grade I or II, or both − patients with cerebral failure alone plus creatinine levels ranging from 1.5 mg/dl to 1.9 mg/dl. ACLF grade 2 includes patients with two organ failures. ACLF grade 3 includes patients with three organ failures or more	Acute hepatic insult manifesting as jaundice (total bilirubin levels of 5 mg/dl or more) and coagulopathy (INR of 1.5 or more, or prothrombin activity of less than 40%) complicated within 4 weeks by clinical ascites, encephalopathy, or both. The severity of ACLF is assessed using the AARC score. The grading system defines Grade 1 by scores of 5–7, Grade 2 by scores of 8–10, and Grade 3 for 11–15
Short-term mortality rate of ACLF according to stratification	By 28 days: Grade 1: 20% Grade 2: 30% Grade 3: 80	By 30 days: two organ failures: 49% three organ failures: 64% four organ failures: 77%	By 28 days: Grade 1: 23% Grade 2: 61% Grade 3: 93%	By 28 days: Grade 1: 13% Grade 2: 45% Grade 3: 86%

The CLIF-C OF score includes sub-scores ranging from 1 to 3 for each of the six components (liver, kidneys, brain, coagulation, circulation, and lungs), with higher scores indicating more severe organ system impairment. Aggregated scores range from 6 to 18 and provide information on overall severity. The AARC score includes sub-scores ranging from 1 to 3 for each of the five components (total bilirubin, hepatic encephalopathy grade, INR, creatinine levels, and blood lactate levels). Aggregated scores range from 5 to 15, with higher scores indicating more severe ACLF.

HBV-related ACLF also needs to be differentiated from HBV-induced Acute liver failure (ALF) (**Table 2**)

**Table 2:** Difference between HBV ALF and ACLF.

Parameters	ALF	ACLF
History	No history of previous liver disease	h/o underlying liver disease
Precipitating factor	Not defined	Always present
Ascites	rarely present	Usually present
Transaminases	15-20 times ULN	3-5 times ULN
Albumin	Usually normal	Mostly low
IgM anti HBc	High	Mostly low
HbsAg	Positive or negative	Positive with high levels
DNA level	Low (< 2.25x10 ^4^ IU/ml)	High (> 2.25x10 ^4^ IU/ml)
HbeAg	Positive in few (25%)	Positive in 50%
Liver Biopsy	features of acute hepatitis with sub massive necrosis of liver	e/o CHB like lymphocytic portal inflammation, interface hepatitis, peri portal fibrosis, bridging fibrosis, and cirrhosis
Endoscopy	No varices	Varices mostly present
Basal core promoter mutation	No	Yes
Pre-core stop codon mutation	No	Yes

## Prevalence of ACLF

It is difficult to provide the exact prevalence of the disease (including HBV-ACLF) due to lack of a single working definition. However, based on hospital registries, approximately 25-40% hospitalized patients with cirrhosis meet the criteria for ACLF (12). Regarding HBV-ACLF, various studies from South East Asia described an incidence rate of around 30-35% of ACLF in patients with underlying HBV-related liver disease. A study from China estimated that the ACLF incidence rate was 2.53 per 100,000 of the general population per year (13, 14).

## Precipitating events

**Figure 1:**
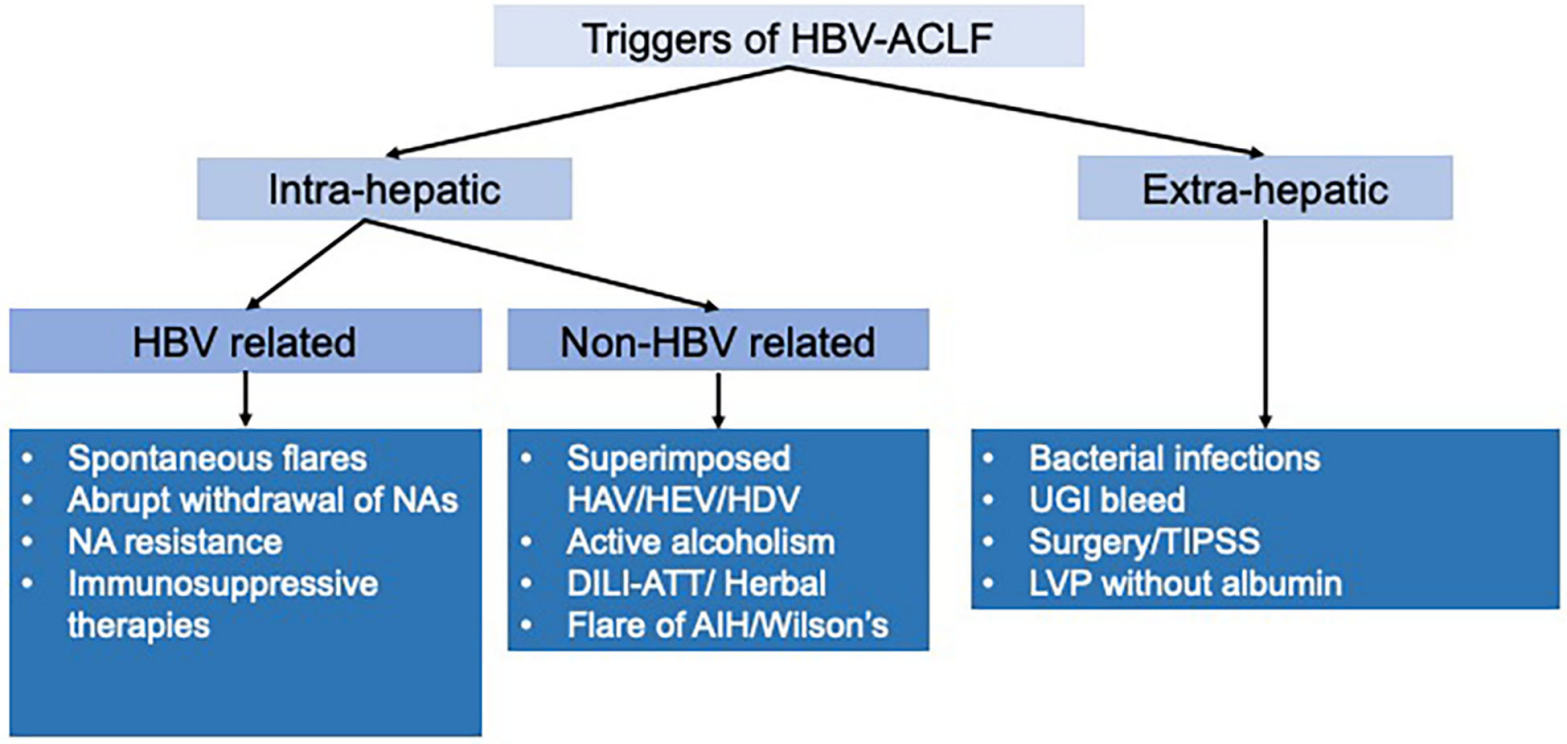
Triggers of HBV-ACLF.

The various precipitating events in HBV-related ACLF can be divided into intra-hepatic or hepatic and extra-hepatic or systemic insults. Intra-hepatic or hepatic insults can be further subclassified into HBV-related and other causes. (**Figure 1**).

While spontaneous flare-ups of hepatitis B are the most common hepatic precipitating factor, accounting for nearly 40%-60% of cases, other important causes include superinfection with other hepatotropic agents, superimposed drug-induced liver injury (DILI), superimposed alcoholic hepatitis, as well as insults such as bacterial infections and GI bleed (15). In extra-hepatic or systemic causes, bacterial infections account for 20%-30% of cases of HBV-related ACLF.

Hepatitis flare is defined as an abrupt elevation of serum alanine aminotransferase (ALT) to over fivefold the upper limit of normal (ULN) (16). This occurs due to immune-mediated killing of the infected hepatocytes. Reactivation of HBV replication can occur spontaneously or following cancer chemotherapy, immunosuppressive therapy after organ transplant, autoimmune disease, or withdrawal of nucleos(t)ide analogues. Hence it is mandatory to screen all such patients with HBV DNA levels. Reactivation can lead to flare-ups of the disease, resulting in acute hepatic failure or ACLF (17). When NUCs with lower barrier to resistance, such as lamivudine, are used, development of resistance to anti-virals is also a significant reason for HBV reactivation leading to ACLF. Flare-ups in such cases can occur due to unchecked replication of drug-induced mutants (18).

## Predisposing factors for development of HBV ACLF

### Host factors

Li et al, have reported higher risk of developing HBV-related ACLF among younger individuals, and among those who have non-cirrhotic HBV (19).

Obese patients are more likely to develop ACLF in general as compared to non-obese individuals, predominantly because of increased risk of renal failure, as was shown among 116704 ACLF patients from the UNOS database (20). In another study from China (>85% patients with HBV-ACLF), both lower and higher BMI categories were associated with higher mortality as compared to the normal BMI categories (21). Data from the AARC group also suggests that obese patients not only have a higher MELD score but also a higher day 30 mortality (6).

Baseline Hepatic venous pressure gradient (HVPG) is an important predictor of mortality in ACLF. The reduction in HVPG significantly influences the outcomes of ACLF patients. In a study of 57 ACLF patients (29% had HBV ACLF), those who survived and underwent paired HVPG measurement demonstrated a significant reduction in HVPG (22).

### Viral factors

Genotype B has been shown to be associated with better short term antiviral response and better survival rate compared to genotype C in patients with HBV-related ACLF (23). The exact reason behind this is not clear but the following few theories have been proposed:(a) Hepatitis B envelope antigen (HBeAg) seroconversion is faster and higher in genotype B as compared to genotype C; (b) Basal core promoter (BCP) mutation, which is more prevalent in genotype C, is a risk factor for progressive liver disease; and (c) Viral suppression is more avid in genotype B as compared to genotype C, and it has been shown that HBV DNA decline at 2 weeks is a predictor of 90-day mortality.

Faster reduction of HBV DNA load holds promise. Garg et al. showed that rapid suppression of HBV DNA in patients treated with antivirals improved the Child Pugh Turcotte (CTP) and MELD scores at 45 and 90 days. Patients with greater reduction in HBV DNA (>2 log 10 IU/ml) at week 2 had lower CTP and MELD scores (24).

Patients with Anti HBe positivity have worse prognosis among HBV-ACLF patients. The reasons might be as follows: (a)These patients have a longer clinical course compared to anti-HBe negative patients, or (b) The rate of cirrhosis was lower in anti-HBe negative group than that in anti-HBe positive. This happens because patients who are anti-HBe positive represent a later stage in the natural history of HBV.

### Type of insult

Among ACLF in general, the outcome also depends on the type of acute injury: intrahepatic or hepatic (reactivation, alcohol, etc) vs extrahepatic or systemic (infections). 28-day mortality remains similar in both the groups but the 90-day and 1-year mortality was better in the group where the acute insult was intra-hepatic or hepatic. Further, among those with intra-hepatic or hepatic insult, the etiology was more likely HBV-related than among those with extra-hepatic or systemic insult (70% vs 56%) (15).

## Pathophysiology

Inflammation, both hepatic and systemic, is the major driver of HBV-associated ACLF. It is an interplay of inflicted injury on host fitness, tolerance, host defense strategies, and dysregulated inflammation. The pathophysiology of ACLF is similar to sepsis. In HBV flare-ups with ACLF, a large number of damage-associated molecular patterns (DAMPs) are released by the dying hepatocytes, which activate the innate immunity through toll-like receptors (TLR). For ACLF due to bacterial infection, pathogen-associated molecular pattern (PAMP) molecules can similarly activate innate immunity. Spontaneous flare-induced ACLF can be complicated with infections from the gut dysbiosis, a leaky gut, or increased bacterial translocation. So, there is an interaction of DAMPs and PAMPs with the innate immune system of the host. This activation of innate immunity leads to the exacerbation of systemic inflammation and release of cytokines, chemokines, oxygen-derived free radicals, eicosanoids, lysosomal and proteolytic enzymes, IL-1, IL-6, IL-18, and tumor necrosis factor-α, leading to systemic inflammatory response syndrome (SIRS). Further, there is fibrin deposition and thrombosis in the microvasculature leading to perfusion abnormality and ischemic hepatocyte dysfunction.

During HBV reactivation, there is an abrupt change in the immunological control of viral replication in a patient with inactive or resolved Hepatitis B. The cross reaction between HBeAg and HbcAg leads to overactive T cells. There is necroinflammation with cellular infiltration, mainly CD81 cytotoxic T lymphocytes (CTLs) directed towards Hepatitis B core antigen (HBcAg). Lai et al. demonstrated that HBV DNA in patients with e-Ag positivity was higher than those with anti-HBeAb positivity. As the disease progresses towards fatality and as liver failure progresses, HBV DNA in HBeAg(+) patients remains stable, but it decreases in anti-HBeAb(+) patients, suggesting that the liver injury in the latter group of patients is more likely to be immune-mediated (25).

## Grading and prognostic scores

A number of prognostic scores have been used for prognostication, guiding management plans, and for assessing response to therapy in ACLF patients. These are:

### General scoring systems

1) Simplified acute physiological score(SAPSII)
2) Acute physiology and chronic health evaluation II (APACHEII)

### Liver specific models

**1)** Child – Turcotte- Pugh score
2) Model for end-stage liver disease, MELD sodium(Na), Initial(i)MELD
3) Kings’ college criteria
4) ACLF research consortium (AARC) score

### Organ failure models

1) Organ system failure score (OSF)
2) Sequential organ failure assessment score (SOFA)
3) Chronic liver failure- sequential organ failure assessment (CLIF- SOFA)
4) CLIF- C- ACLF score
5) NACSLED score

Of these, the CLIF C ACLF score and the AARC score are important. Although these scores were originally described for a general ACLF population (and not specifically designed for HBV-ACLF), a significant proportion of patients in the original descriptions of the AARC score had HBV as the etiology.

#### The CLIF C ACLF score

The CLIF C ACLF score combines the degree of organ failure of both the hepatic and extra hepatic components along with age and total leucocyte count. It gives an assessment of the overall physiological state and correlates well with the prognosis (26). According to the CLIF consortium, ACLF can be graded as Grade 1, 2, or 3, based on the degree and number of organ failures present.

#### AARC score and ACLF grade

The AARC score has been proposed by the APASL consortium in their group of patients. It has been shown to predict short-term mortality (27). It has been proven to be superior to MELD/MELD Na, CLIF-SOFA, and SOFA scores for patients with ACLF.

Both these and other scores dynamically aid in differentiating patients into those that will or will not survive without definite therapeutic interventions like liver transplantation (LT). A trend of AARC score within the first week can predict the need of liver transplant. Scores of< 10 at presentation or decreases in score below 10 by the end of first week are associated with a higher chance of survival. Patients with AARC Score ≥ 11 should be listed for urgent LT.

A study by Li et al, compared the performance of several previously described prognostic scores, specifically in HBV-related ACLF. They found that the AUROC of CLIF-Organ failure (CLIF-OF) score (AUROC:0.906) was superior to that of MELD (AUROC: 0838), CLIF-SOFA (AUROC: 0.876), and CLIF-C ACLF (AUROC: 0.858) scores (28).

Another study compared eight prognostic scoring systems in 249 ACLF patients (91 of whom had HBV-related ACLF). CLFI-OF, CLIF-C ACLF, and APACHE III outperformed other models in the entire cohort and in the subgroup of HBV-related ACLF (29).

### Prognostic scores specifically designed for HBV ACLF

There are certain scores that have been specifically designed for patients who have HBV-related ACLF. A dynamic model designed for HBV-related ACLF treated with nucleoside analog was described by Lin et al (30)

This is based on the following equation:


$$\begin{array}{l} R=\;0.94\text{ x }Bilirubin\;+\;0.53\text{ x }evolution\;of\;bilirubin–\;\\ 0.45\text{ x }PT-A\;–\;0.22\text{ x }evolution\;in\;PT-A\;–\;0.1\text{ x }PLT\;+\;10\text{ x }anti\;HBe. \end{array}$$

This model was found to be superior to MELD, MELD-Na, and CLIF-SOFA in predicting 9day mortality. Also, it is the first dynamic model for HBV-related ACLF treated with NUCs. Similarly, other scores developed from routine clinical parameters (Age, bilirubin, INR, AFP and Platelet counts, urea, neutrophil counts, and HE) also give good discrimination of 28- and 90- day survival (31, 32).

TPPM (Tongji prognostic predictor model) incorporates total bilirubin, INR, HBV DNA, and complications as parameters. It could better predict 90-day mortality than MELD and MELD-Na in HBV ACLF. However, these are mostly single center-derived criteria that need validation in multicenter and multinational cohorts (33).

A recent study also compared the prognostic performance of NACSLED and EASL-CLIF criteria in diagnosing ACLF. Of their cohort, 65% had HVB-related ACLF. They found that there was no difference between the two criteria in predicting 7 and 90-day survival/mortality. In addition, both the criteria performed equally well in HBV and non-HBV cohorts (34).

## Outcomes

ACLF has high short-term mortality. Data from the CANONIC study showed an overall 28-day mortality in 33% of all cases of ACLF (4). Liver and coagulation failure were more common types of organ failure in HBV ACLF, whereas renal and cerebral failure were seen more commonly in non-HBV ACLF. Another interesting observation was that kidney failure rate in the HBV patients was significantly lower (28.6% vs. 52%). The mechanism of lower rates of renal failure in the HBV ACLF population is not clear (18). The short-term mortality (28/90 days) was much higher in the patients who developed sepsis or received renal replacement therapy or mechanical ventilation. Multiorgan failure was the most common cause of death. In patients with HBV ACLF, the type of acute insult (hepatic or extra hepatic) did not have an impact on mortality. The short term(28-days) mortality was 48.3% and 50.7% respectively in cases of hepatic or extrahepatic insults (15).

## Treatment

### Supportive therapy

All patients need intensive care and close monitoring. These patients should preferably be admitted to a transplant center. Early identification and treatment of the precipitating factors if possible is the most important initial treatment, for example, control of upper gastrointestinal (UGI) bleed, withdrawal of toxic drugs, or control of infection. One should have a low threshhold for starting antimicrobial therapy. Various common infections in these patients are spontaneous bacterial peritonitis, bacteremia, pneumonia, and urinary tract infection. The challenge is to choose the most appropriate antibiotic regimen which should be guided by the local microbiological profiles prevalent in individual regions/centers. Use of albumin has a doubtful role in improving the intravascular volume and preventing infection and acute kidney injury. Various other organ supports should be provided in the form of renal replacement therapy, inotropes, and ventilatory support.

### Antibiotic therapy

Sepsis is not only an important precipitant of ACLF among patients with underlying chronic liver diseases, but also a significant complication among patients who develop ACLF. In fact, sometimes it is impossible to dissect if infection led to ACLF or was an outcome among patients seen for the first time as ACLF. Bacterial infections can complicate the course of one third to one half of ACLF patients and have been associated with higher incidence of organ failures and higher mortality (4, 9, 35). The most frequent infections encountered in such patients are spontaneous bacterial peritonitis (SBP), pneumonia, urinary tract infections (UTIs), and spontaneous bacteremia. For patients presenting with ACLF, all surveillance cultures should be sent. In cases of suspected sepsis, it is recommended that empirical broad-spectrum antibiotics should be started preferably before the development of sepsis (golden window) (6, 36).. The choice of antibiotics should be based on the local antibiotic sensitivity patterns of the prevalent strains. The role of antifungals in the early phase is debatable and still a matter of investigation.

### Anti-viral therapy

APASL guidelines strongly recommend the early and prompt use of antiviral therapy in HBV ACLF. The aim is to rapidly decrease the viral load, leading to reduced hepatocyte cell death. Rapid reduction of viral load has been shown to improve the survival rate. An important study from India demonstrated that 2 log decrease in HBV DNA at week 2 improved survival. It also prolongs the time to LT and improves transplant outcomes (37).

*Peg IFN-α* is contraindicated in patients with ACLF as it may worsen the hepatitis flare-ups due to immune-mediated killing of hepatocytes.

#### Nucleos(t)ide analog

Drugs with potent antiviral efficacy and a high barrier to resistance are preferred. **Table 3** summarizes the studies of NUCs among patients with HBV-ACLF.

**Table 3:** NUCs in HBV ACLF.

Reference	Study design	Drug	No. of patients	Survival benefit
Sun et al (38)	Retrospective cohort	LAM	130	No
Chen et al (36)	RCT	LAM or ETV	42 ETV vs 30 LAM	Yes with ETV
Qin et al (39)	RCT	Telbivudine	12	Yes
Yang et al (40)	RCT	ETV(0.5)	55	Yes
Garg et al (37)	RCT	Tenofovir vs placebo	14vs 27	yes

##### Lamivudine

Multiple investigators, including Chan et al (41), have used lamivudine as monotherapy in this group of patients. However, no encouraging results were obtained. It did not prevent progression to hepatic failure nor was there any survival benefit. One third of the patients developed lamivudine resistance and virologic breakthrough, which might have been responsible for the lack of survival benefit.

##### Adefovir

It has a relatively weak antiviral activity and slow onset of action. Hence, it is not recommended as first-line therapy in the presence of ongoing ACLF.

##### Entecavir

Chen et al, showed that ETV rapidly reduced the HBV replication, however MELD score and liver function showed no significant change (42).Lai et al. showed that, among HBeAg negative patients with ACLF, entecavir did not show any superiority over lamivudine. There was no difference in the virologic and biochemical response or deterioration rate (43). Further, Entecavir should be avoided in the presence of renal dysfunction and MELD>24 as it may cause lactic acidosis in this group of patients.

##### Tenofovir

Garg et al, from India showed that tenofovir disoproxil fumarate(TDF) significantly decreased the HBV DNA load in 2 weeks and improved the MELD and CTP score. It also improved the survival rate (37). Since renal damage is known to occur in ACLF, and TDF is reported to cause kidney injury, it could be replaced by the newly approved tenofovir alafenamide (TAF) in patients with acute kidney injury (AKI) (44). However, data suggests that TAF and TDF may have similar efficacy and safety in HBV-related ACLF (45).

### Liver transplantation

Liver transplantation is the only definitive therapy for HBV-related ACLF in patients who do not stabilize on medical management. All patients admitted with ACLF should be evaluated for LT. APASL consensus agreed with the King’s college hospital criteria for listing for LT. Unfortunately, no extra weightage is given to ACLF in the MELD system despite its high mortality rate and very short window of opportunity. Right lobe living donor transplant is an attractive option for HBV ACLF patients. The immediate post-operative period can be difficult for these patients. 1-year and 5-year survival rate is comparable with non-ACLF patients (46). Among the 238 patients, a 5-year post-LT survival of >80% was demonstrated. Data from the CANONIC study shows that 9% of patients satisfying the ACLF criteria could be transplanted within 28 days and 15% within 90 days after admission. In patients with ACLF grade 2 or 3, survival without LT was<20%, which increased to 80% at 1 year in those who received LT. The results were comparable with those patients who were transplanted without ACLF for other end-stage liver diseases. The time lag between ACLF diagnosis and LT was 11 (1–28) days (4). The long-term survival is excellent, with a 5-year survival rate of >70% demonstrated even in patients with high MELD scores (47).Among patients with HBV-ACLF, Peri-operative intravenous hepatitis B immunoglobulin (HBIG) combined with post-operative nucleos(t)ide analogue (NA) can reduce HBV recurrence (48). However, the role of HBIG in prevention of post-transplant HBV recurrence is declining and many centers have either started using low-dose regimens or have totally given up the post-operative use of HBIG (49).

However, less than one fourth of the patients are fortunate enough to go through LT. Some patients cannot receive transplants due to their advanced age, active alcoholism, sepsis, other associated serious co-morbidities, or psychological factors. Sometimes the patient is too sick, making them unfit for transplant. Hence the timing is very crucial, as there is a very short window for LT opportunity. Furthermore, it is an expensive therapy and there is a shortage of donor organs.

### Liver support systems

Since most of these patients have advanced liver failure and many cannot undergo liver transplantation, liver support systems may offer a bridge or therapeutic option. Xiao et al, carried out a retrospective propensity score-matched study of 790 HBV ACLF patients and found that, compared to standard medical therapy (SMT; n=412), the Artificial liver support system(ALSS; n=378) improved the 28-day (65.2% vs 59%; p=0.04) and 90-day survival rates (51% vs 42.3%; p=0.01) and laboratory parameters in HBV-ACLF patients. In the ALSS group, the patients either received plasma exchange or CRRT. However, as can be seen, the difference was not much and we need more data from well conducted and controlled studies before this form of therapy can be routinely recommended (50)

#### MARS

Molecular adsorbent recirculating system is a non-biological dialysis-derived technique which supports the detoxification function of the liver. It effectively decreases the bilirubin and improves coagulopathy and encephalopathy, but does not offer any survival advantage (51).

#### Plasma exchange

Hepatocyte injury during ACLF leads to release of DAMPs in the plasma, which trigger innate immune responses leading to further exacerbation of liver injury. In addition, there are gut-derived PAMPs, cytokines, and high levels of ammonia and bilirubin in the plasma of liver failure patients. It has been postulated that these molecules may impair liver regeneration. Therefore, replacement of the patient’s plasma with fresh frozen plasma may aid in the regeneration of the liver or reverse the ongoing liver injury and lead ultimately to recovery. High-volume PE has shown to modulate the inflammatory cytokine storm, dampen the anti-inflammatory responses, and ameliorate multiorgan failure, which resulted in improved transplant-free survival in a randomized control trial in 182 patients with ALF (53). Studies show that it holds promise in either increasing the survival or reducing the MELD score. Please refer **Table 4**. In one Chinese study, 62 patients with HBV ACLF who received PE treatment were compared with 131 patients treated with standard care. The 30-day survival rate of the patients who received PE was significantly higher compared to controls (41.9 versus 25.2%). Interestingly, this benefit was limited only to patients with less severe disease with MELD scores in the range of 20–30 and was not seen for patients with MELD scores >30. Unfortunately, it is this very group of patients (those with higher MELD scores) who are actually in need of this kind of therapy (54).

**Table 4:** Plasma exchange for ACLF from HBV reactivation (52).

Studies	Benefit
Mao et al	30 days survival 50%vs 31.7%
Ling et al. (2012)	Reduced MELD prior to LTx
Wan et al. (2015)	12 weeks survival 29% vs 14%

In a study by Wan Yue Meng and colleagues, the efficacy of PE in patients with ALCF and acute decompensation of cirrhosis treated with entecavir (ETV) was investigated. A 3-month follow-up showed better survival in patients who received plasma exchange. Not much difference was seen in the HBV DNA load between the two arms. However, it should be noted that this was a retrospective study (55).

Although these studies from China do suggest that PE and ALSS may have some benefit among HBV-related ACLF, similar results have not been obtained in ACLF of other etiologies. However, even in the HBV-related ACLF groups we do need data from large prospective multinational controlled studies before ALSS or PE can become the standard of care in these patients. Until then, this form of therapy should best be viewed as a bridge to LT.

### Other novel therapies

#### Granulocyte colony stimulating factor

This therapy mobilizes bone marrow-derived stem cells. Initial research holds promise by demonstrating reduced short-term mortality. It also decreases the risk of hepatic encephalopathy, hepatorenal syndrome, and sepsis.

Duan et al. in 2013 studied 55 patients with HBV ACLF. They subjected 27 patients to G-CSF and standard care and 27 patients to standard care alone. They showed that the peripheral neutrophil and CD34+ cell counts in the G-CSF group increased on day 3 and continued to rise on day 7; it remained elevated on day 15 compared to those of the control group. There was improvement in the CTP and MELD score of the patients who received GCSF. After 3 months of follow-up, the survival rate in the treatment group (48.1%) was significantly higher than that in the control group (21.4%) (56).

Similarly, Garg et al. showed that G-CSF therapy more than doubles the percentage of patients with ACLF who survive for 2 months; it also significantly reduces CTP, MELD, and SOFA scores and prevents the development of sepsis, hepatorenal syndrome, and hepatic encephalopathy, especially in patients with severe alcoholic hepatitis and reactivation of hepatitis B (57). However, a further study by Engelmann et al., which was a multi-center randomized trial, showed 176 patients of ACLF failed to demonstrate a significant benefit with granulocyte-Colony Stimulating Factor (G-CSF). The use of G-CSF neither improved 3- nor 12-month transplant-free survival nor did it lead to improvement in MELD scores or new infections (58). Several studies from Southeast Asia show this limitation can be due to differences in patient cohort and their pathophysiology. It also varies on the basis of ACLF defining criteria, as APASL does not include extra-hepatic insults in its definition (59).

Until more data is made available that unequivocally proves its benefit, G-CSF therapy should be regarded as experimental for the treatment of HBV ACLF and ACLF due to other etiologies.

#### Stem cell therapy

Hepatocyte transplant, mesenchymal stem cells, or stromal cells are also emerging therapies. These pluripotent cells are extracted from the umbilical cord, placenta, or bone marrow. They have the ability to grow into hepatocytes. A meta-analysis of 12 studies using mesenchymal stem cells demonstrated that there was an improvement in MELD score, serum albumin levels, and coagulation parameters, but no impact on survival. Apart from fever, no other adverse event was noted (60). A recent study from China evaluated hepatic artery infusion of peripheral blood stem cells after mobilization with G-CSF among HBV-related ACLF patients. The 90-day survival was higher in the stem cell therapy groups as compared to PE alone or PE with G-CSF (85% vs 50% vs 65%; p=0.03) (61). These early encouraging results on the use of stem cells in HBV-related ACLF need to be validated in large trials before these results can be applied widely.

In summary, ACLF is a distinct syndrome and HBV-related ACLF has some unique characteristics that have been discussed in detail above. Timely application of NAs can salvage a proportion of patients with ACLF. Others will progress to a stage where liver transplantation will become inevitable. ALSS, especially PE, can act as a bridge and may even help in the reversal of the syndrome in a minority of patients. All other therapies, including stem cell therapy, are considered experimental until more robust data is made available. Until more effective therapies become available, the short- and long-term mortality for people with this syndrome will remain high.

## Author contributions

PG has made a substantial contributions to the concept of the article. Drafted the introduction, Definition, Prevalence of ACLF, Precipitating events and Pathophysiology. KM Revised the article critically for important intellectual contents and drafted the Grading and prognostic scores, outcomes and treatment. All authors contributed to the article and approved the submitted version.
